# Unmet support and information needs of relatives of patients with advanced or metastatic breast cancer: results of the BRE-BY-MED study

**DOI:** 10.1007/s00520-026-10672-4

**Published:** 2026-04-17

**Authors:** Lilly Sophia Brandstetter, Anna Grau, Johannes Löffler, Max Müller-Reiter, Jessica Salmen, Jens-Peter Reese, Peter Heuschmann, Achim Wöckel

**Affiliations:** 1 Institute for Clinical Epidemiology and Biometry, Julius-Maximilian University Würzburg, Würzburg, Germany; 2 Department of Gynaecology and Obstetrics, University Hospital Würzburg, Würzburg, Germany; 3 Faculty of Health Sciences, Technische Hochschule Mittelhessen University of Applied Sciences, Giessen, Germany; 4 Institute of Medical Data Science, University Hospital Würzburg, Würzburg, Germany; 5 Clinical Trial Centre Würzburg, University Hospital Würzburg, Würzburg, Germany

**Keywords:** Metastatic breast cancer, Caregivers, Relatives, Unmet needs, Information needs, Quality of life

## Abstract

**Purpose:**

Cancer often affects not only the patients but also their relatives, as diagnosis and prognosis, treatment, and side effects can have a significant psychosocial impact. We aimed to investigate unmet information and support needs and quality of life in a German cohort of relatives of metastatic breast cancer (mBC) patients.

**Methods:**

Between July 2022 and August 2023, adults with mBC were included in the BRE-BY-MED “Breast Cancer Care in Bavaria for Patients with Metastatic Disease” cohort study (DRKS00026601) at the University Hospital Würzburg. Relatives of these patients completed a questionnaire on unmet support and information needs at the 3-month follow-up.

**Results:**

75.8% (*n* = 25) of the relatives had at least one unmet support need, mostly observed in the categories “medical information about the relative’s illness” (54.5%) and “contact person who coordinates the treatment/aftercare” (42.4%). 45.5% (*n* = 15) of the relatives had at least one information need, with the highest needs in the categories “the likely course of the disease in the relative” (39.4%) and “how to deal with the treatment side effects” (36.4%). In 72.7% and 88.0% of the relatives, respectively, there was evidence for severe depressive or anxiety symptoms.

**Conclusion:**

Dedicated support programmes for relatives of patients with mBC should be developed, targeting their unmet support and information needs regarding the disease, treatments, side effects, prognosis, and aftercare. Furthermore, physicians should be aware of actively involving mBC patients’ relatives in the disease and treatment process.

## Introduction

Metastatic breast cancer (mBC) is defined as breast cancer (BC) that has spread to other parts of the body. mBC is newly diagnosed in about 10% of all cases, and about 30% of all patients develop an mBC within the course of their disease [1, 2]. mBC is incurable with a median survival time between 2 and 4 years [1, 2]. The focus of treatment in mBC is on prolonging patient survival, providing adequate symptom management, and promoting quality of life (QoL) [1–3].

Cancer often affects not only the patients but also their relatives [4–6]. Relatives are often expected to provide complex care at home with little preparation or support. This can include watching for and helping with treatment side effects, administering medication, and organising doctor’s visits [7]. Furthermore, the diagnosis, treatment, and its side effects, as well as the prognosis, can have a significant psychosocial impact on the patients’ relatives, often characterised by worry and anxiety [4, 8]. A previous study has shown that relatives of palliative patients generally have a lower quality of life (QoL) than relatives of patients receiving curative care [9]. According to the current definition of palliative care provided by The World Health Organization (WHO), comprehensive care for patients with advanced cancer should include treatment as well as qualified support offered to their family members, with the aim of improving the QoL of both patients and their families [10].

Various studies on unmet needs of relatives and their QoL have been conducted worldwide, based on patient populations with very different diseases. Lambert et al. (2012) summarised 29 studies that evaluated unmet needs of cancer patients’ caregivers in a systematic literature review [11]. They found that the standardised mean number of reported unmet needs in the included studies ranged between 5 and 47% and that 15.5–67.9% of the relatives experienced at least one unmet need. Particularly, high numbers of unmet needs were reported by relatives of partners in palliative care, such as those with metastatic cancers.

A study from Iceland on 223 relatives of cancer patients (various stages) receiving chemotherapy not only assessed the needs in terms of caring for their loved one, but also the QoL, anxiety, and depression of the relatives [12]. In this study, a low to moderate correlation was found between the number of satisfied important needs and a higher QoL. The highest prevalence of unmet needs was about 55%.

To date, there is no data on the prevalence of information and support needs among relatives of patients with mBC in Germany. Therefore, the primary aim of the present study was to investigate unmet information and support needs in a German cohort of relatives of mBC patients. Secondary aims were to assess the QoL of the relatives and to analyse the relation to the information and support needs.

## Methods

### Study design

Data are derived from patients and their relatives participating in BRE-BY-MED “Breast Cancer Care in Bavaria for Patients with Metastatic Disease” (DRKS00026601). BRE-BY-MED was a prospective cohort study at the Department of Gynaecology and Obstetrics of the University Hospital Würzburg with the primary aim to estimate the prevalence of guideline adherence [13].

### Inclusion and exclusion criteria

BRE-BY-MED included patients of both sexes, with prevalent or newly diagnosed advanced or mBC (hereafter referred to as mBC), aged ≥ 18 years, living in Bavaria, and who gave written informed consent. Advanced or mBC was defined by ICD-10 Code C50 or C76-C80 and TNM classification pTx-pNx-M1 or pTx-pN(1–3)-Mx. Exclusion criteria are minimised to age (< 18 years), disease (non-advanced or non-mBC), and not living in Bavaria to guarantee a most representative study population of clinical routine care. For the participating relatives, no inclusion or exclusion criteria were defined.

### Ethics approval and consent to participate

The study was performed in full accordance with the principles of the “Declaration of Helsinki” (as amended in Tokyo, Venice and Hong Kong). All methods of the study were fully approved by the ethics committee of the Medical Faculty of the University Wuerzburg (reference number 137/21). Written informed consent was obtained from all patients. As no person-related data were collected from the relatives and the survey was anonymous, no informed consent was required. The responsible data protection officer accepted the data management concept.

### Data collection

In July 2022, we conducted a pilot study to assess the feasibility of the study design. Data were collected at baseline (study inclusion) and after a 1-month follow-up period. Between September 2022 and August 2023, mBC patients were informed by the project staff about the BRE-BY-MED study and asked to participate. After written informed consent was obtained, patients completed the baseline survey comprising information on sociodemographic factors (i.e. age and sex).

#### Clinical data

Information on diagnosis and therapies was manually extracted from routine medical records of the Department of Gynaecology and Obstetrics of the University Hospital Würzburg and was entered into an electronic case report form (eCRF) by study personnel (LB), trained by an experienced physician of the Department of Gynaecology and Obstetrics of the University Hospital Würzburg (MMR). Clinical data comprised information on the diagnosis (ICD-10 code), the type of systemic mBC therapy, other therapies (surgery, radiotherapy), comorbidities (Charlson Comorbidity Index (CCI)), menopausal status, hormone receptor (HR), and human epidermal growth factor receptor 2 (HER2) status.

#### Unmet support and information needs and mental health of patients

Three months after study inclusion, patients were sent a follow-up questionnaire on anxiety and depressive symptoms, information, and support needs. Anxiety and depressive symptoms were assessed using the PHQ-4 (Patient Health Questionnaire 4) [14]. The PHQ-4 consists of the PHQ-2, which measures depressive symptoms, and the GAD-2 (Generalized Anxiety Disorder 2), which measures anxiety symptoms. For the present analysis, summary scores of both scales were dichotomised using the established cut-off score of ≥ 3 points, equalling moderate to severe anxiety and depressive symptoms, respectively [14]. Patients’ unmet information needs were assessed in eight different aspects of mBC therapy (i.e. diagnosis, likely course of the disease, treatment options, risks/side effects, nutrition counselling, coping mechanisms, psychological support, possibility of follow-up care) and measured on a 5-point Likert scale, ranging from 1 = feeling not informed to 5 = feeling fully informed. For each aspect, patients could further state whether they wished for more information or not. Patients’ unmet support needs were assessed in six healthcare areas (i.e. medical care, social support, counselling/information, emotional support, help with daily activities, social or financial counselling) and measured on a 5-point Likert scale, ranging from 1 = no need for support to 5 = high need for support.

#### Unmet support and information needs and mental health of relatives

Together with the patient survey at 3 months after study inclusion, patients were sent a questionnaire for a relative. Patients were asked to forward this questionnaire to a relative of their choice. All relatives who completed and returned the questionnaire were included in the study.

The questionnaire for the relatives included sociodemographics (age group, sex, degree of kinship, scholar education level), anxiety and depressive symptoms, unmet support and information needs, the relevance of different information sources for information acquisition, and questions on how helpful different contents of a potential course or app would be for them. Degree of kinship was assessed in three categories: (I) spouse/partner, son/daughter, brother/sister, son-in-law/daughter-in-law, parent; (II) uncle/aunt, cousin, nephew/niece, grandchild; and (III) none of the above/other. Anxiety and depressive symptoms were assessed as described for the patient survey; see the “Unmet support and information needs and mental health of patients” section.

The unmet support needs were assessed in ten different aspects of mBC therapy (i.e. recommendations on health-related behaviour, counselling on partnership/family, opportunities to talk with other people, contact with people in the same situation, medical information about the relative's illness, contact person who coordinates the treatment/aftercare, social or financial counselling, help with household/daily activities, transport to appointments related to the relative's cancer) measured on a two-point scale. Relatives either state that they do not need support because they report having no problems in the respective aspect, or because they are already receiving support. Alternatively, they state that they have low, medium, or high support needs. The information needs were assessed in nine different aspects of mBC therapy (i.e. diagnosis, likely course of the disease, examinations/tests carried out, treatment options, risks/side effects and how to deal with them, alternative/complementary medicine, signs of a recurrence of the disease, how to deal with the stress on the family/partnership as a result of the disease, possibility of follow-up care) measured on a 5-point Likert scale, ranging from 1 = feeling not informed to 5 = feeling fully informed. For each aspect, relatives could further state whether they wished for more information or not.

The relevance of information sources for information acquisition included print media, digital media, professional counselling, and support groups. The relevance for each information source was measured on a 6-point Likert scale, ranging from 1 = very important to 6 = not important. In addition, relatives were asked about their satisfaction with the level and the amount of received/obtained information, measured on a 4-point Likert scale, ranging from 1 = very dissatisfied to 4 = very satisfied.

Furthermore, relatives were asked how helpful different contents (i.e. exercises for personal coping with the new situation, relaxation exercises, information on socio-legal issues) of a potential course or app would be for them. The helpfulness for each content was measured on a 6-point Likert scale, ranging from 1 = not helpful at all to 6 = very helpful.

The prevalence of unmet needs was defined as “need for support” (low, medium OR high) OR “Information need” (yes) (questionnaire modified according to Faller [15].

### Statistical analyses

Descriptive statistics were computed for continuous data (mean, ± standard deviation (SD) or median, interquartile range (IQR)) and categorical data (sample size and percentage).

To better classify the mental health outcomes of relatives, the proportion of relatives reporting moderate to severe anxiety or depressive symptoms was compared with values from the general population in Germany. For this purpose, data from male participants of the Mental Health Surveillance Study conducted by the Robert Koch Institute were used [16, 17]. In parallel with the 3-month follow-up of the present study (December 2022–November 2023), about 19% and 11% of the participants exhibited moderate to severe depressive and anxiety symptoms, respectively.

Subgroup analyses for factors associated with relatives’ unmet needs and information needs were performed descriptively for age, educational level, depressive and anxiety symptoms, and knowledge of the disease. Patient-related factors included in the subgroup analysis were CCI, menopausal status, HR/HER2 status of the tumour, time since first diagnosis of metastasis, type of systemic mBC therapy, depressive and anxiety symptoms, and their unmet and information needs. For the subgroup analyses, only information from patients whose relatives participated in the study was included.

## Results

### Study recruitment

**Fig. 1:**
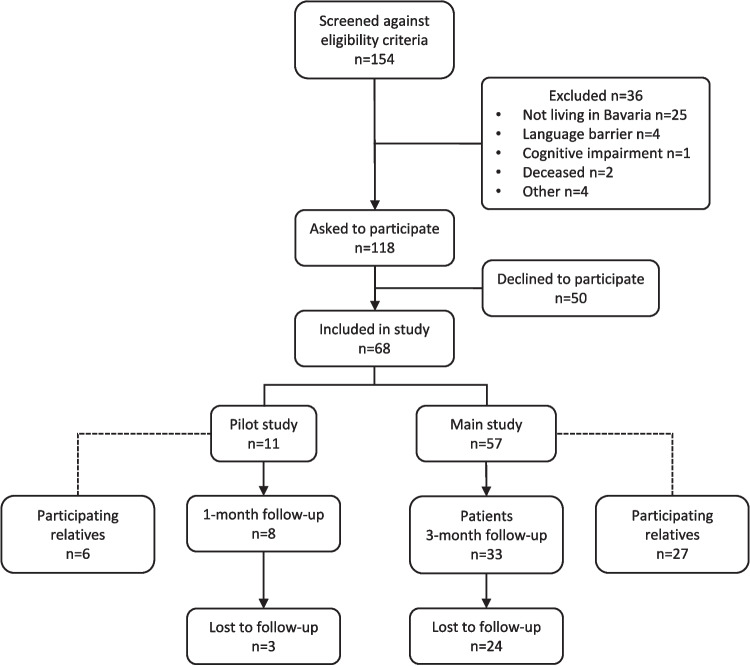
Flowchart of the BRE-BY-MED study

A total of 68 patients were consecutively enrolled in the BRE-BY-MED study, of which 11 were included in the pilot study and 57 in the main study. In the pilot study, eight patients provided information at 1-month follow-up (response rate 72.2%). In the main study, 33 patients provided information at the 3-month follow-up (response rate 57.9%). In total, 33 relatives took part in the survey at the 1–3-month follow-up, respectively (response rate 48.5%). The study flowchart is presented in Fig. 1.

### Characteristics of the participating patients at baseline

The median age of the participating patients was 57.5 years (IQR = 48.0–64.8 years), and one patient was male. Most patients had a CCI of 1–2 points (*n* = 54, 79.4%). 20.6% (*n* = 14) had a CCI of ≥ 3 points. Most patients were postmenopausal (*n* = 46, 67.6%; premenopausal: *n* = 18, 26.5%; perimenopausal: *n* = 1, 1.5%; unknown menopausal status: *n* = 1, 1.5%). Furthermore, most patients had HR + (*n* = 52, 76.5%; HR-: *n* = 16, 23.5%) and HER2- (*n* = 53, 77.0%; HER2 + : *n* = 15, 22.1%) tumours. About half of the patients received their first diagnosis of metastasis within less than a year before study recruitment (*n* = 36, 52.9%; time since first diagnosis of metastasis ≥ 1 year: *n* = 32, 47.1%). Concerning the mBC therapy, 51.5% (*n* = 35) received chemotherapy, 58.8% (*n* = 40) endocrine therapy, 52.9% (*n* = 36) osteoprotective therapy, 32.4% (*n* = 22) targeted therapy, and 1.5% (*n* = 1) immunotherapy. In 26.5% (*n* = 18; missing *n* = 1) and 19.1% (*n* = 13; missing *n* = 1) of the patients, respectively, there was evidence for severe depressive or anxiety symptoms according to the cut-off of ≥ 3 points for both scales. 38.2% (*n* = 26) of the patients reported in at least one category that they felt slight or not informed. 51.5% (*n* = 35; missing *n* = 3) of the patients reported at least one unmet need. The detailed description of the study population was published elsewhere [18].

### Characteristics of the participating relatives at 3-month follow-up

Most of the participating relatives were male and were aged ≥ 50 years (Table 1). The distribution across the categories of the highest educational level was balanced. All relatives fell into the degree of kinship category I. 66.7% (*n* = 22) of the relatives had other close persons (e.g. family, friends) with an advanced or metastatic cancer in the past, and 51.5% (*n* = 17) of the relatives obtained information regarding advanced or metastatic cancers (e.g. by reading books, speaking to people affected). In 72.7% and 88.0% of the relatives, respectively, there was evidence for severe depressive or anxiety symptoms according to the cut-off of ≥ 3 points for both scales.

**Table 1:** Characteristics of the relatives

	All relatives (*n* = 33)
*Age group (n, %)*	
18–34 years	1 (3.0)
35–49 years	5 (15.2)
50–64 years	13 (39.4)
≥ 65 years	14 (42.4)
*Male sex (n, %)*	26 (78.8)
*Highest educational level (n, %)*	
Secondary modern school (Hauptschule)	8 (24.2)
Intermediate school/O-levels (Realschule)	10 (30.3)
A levels/university entrance qualification (Fachhochschulreife/Abitur)	14 (42.4)
	*missing n* = *1*
*Degree of kinship (n, %)*	
Category I: spouse/partner, son/daughter, brother/sister, son-in-law/Daughter-in-law, parent	33 (100)
Category II: uncle/aunt, cousin, nephew/niece, grandchild	0 (0)
Category III: none of the above/other	0 (0)
*PHQ-2*	
Summary score (median, IQR)	4.0 (3.0–4.0)
Severe depressive symptoms (*n*, %)	24 (72.7)
	*missing n* = *2*
*GAD-2*	
Summary score (median, IQR)	3.0 (3.0–4.0)
Severe anxiety symptoms (n, %)	29 (88.0)
	*missing n* = *1*

### Unmet support and information needs of relatives

75.8% (*n* = 25) of the relatives had at least one unmet support need (Table 2). Most support needs were observed in the category “medical information about the relative’s illness” (54.5%) and “contact person who coordinates the treatment/aftercare” (42.4%).

**Table 2:** Unmet support and information needs of the relatives

	All relatives (*n* = 33)
*Support need on (n, %)*	
Recommendations on health-related behaviour	12 (36.4)
Counselling on partnership/family	13 (39.4)
Opportunities to talk with other people	9 (27.3)
Contact with people in the same situation	12 (36.4)
Medical information about the relative's illness	18 (54.5)
Contact person who coordinates the treatment/aftercare	14 (42.4)
Social or financial counselling	13 (39.4)
Help with household/daily activities	12 (36.4)
Transport to appointments related to the relative's cancer	10 (30.3)
Other (i.e. doctor-relative consultation, support from the employer)	2 (6.1)
	*Missing n* = *1*
*Information need on (n, %)*	
The diagnosis of the relative	6 (18.2)
The likely course of the disease in the relative	13 (39.4)
The examinations/tests carried out	7 (21.2)
The various treatment options	8 (24.2)
The risks/side effects of the treatment	7 (21.2)
Alternative/complementary medicine	11 (33.3)
The signs of a recurrence of the disease	10 (30.3)
How to deal with the treatment side effects	12 (36.4)
How to deal with the stress on the family/partnership resulting from the disease	9 (27.3)
The possibility of follow-up care	10 (30.3)
Other (i.e., opportunity for doctor-relative consultation)	1 (3.0)

Furthermore, 45.5% (*n* = 15) of the relatives had at least one unmet information need (Table 2). The highest information needs were in the category “The likely course of the disease in the relative” (39.4%), “how to deal with the treatment side effects” (36.4%).

### Relevant information sources and satisfaction with the information received

The relevance of different information sources for information acquisition for the relatives is depicted in Table 3. Relatives mostly relied on professional counselling. However, print and digital media were the second most important information sources.

**Table 3:** Relevance of information sources for information acquisition for relatives (n = 33) (n, %)

Information source	1 = very important	2	3	4	5	6 = not important
*Print media*	13 (39.4)	5 (15.2)	5 (15.2)	3 (9.1)	2 (6.1)	5 (15.2)
*Digital media*	9 (27.3)	5 (15.2)	2 (6.1)	3 (9.1)	3 (9.1)	9 (27.3)
*Professional counselling*	20 (60.6)	10 (30.3)	3 (9.1)	0 (0)	0 (0)	0 (0)
*Support groups*	2 (6.1)	7 (21.2)	2 (6.1)	10 (30.3)	10 (30.3)	2 (6.1)
*(Missing n* = *1)*						

Regarding the satisfaction with the level of received/obtained information, most relatives stated to be moderately (*n* = 9, 27.3%) to well (*n* = 19, 57.6%) satisfied. Only three relatives reported being very dissatisfied (9.1%). Yet also only two relatives stated to be very satisfied (6.1%).

Regarding the satisfaction with the amount of information received/obtained, most relatives stated to be moderately (*n* = 15, 45.5%) to well (*n* = 13, 39.4%) satisfied. Only three relatives reported being very dissatisfied (9.1%). But also only two relatives stated to be very satisfied (6.1%).

### Factors associated with unmet needs and information needs of the relatives

Relatives in older age groups and with higher educational levels were less likely to report unmet needs or information needs compared to their counterparts (Table 4). Conversely, relatives who had close persons with an advanced or metastatic cancer in the past, who obtained information regarding advanced or metastatic cancers, where patients had HER2- tumours, and where patients had at least one unmet or information need, were more likely to report unmet needs or information needs compared to their counterparts.

**Table 4:** Factors associated with unmet needs and information needs of the relatives (n, %)

	Unmet support needs		Unmet information needs	
	At least one (*n* = 25)	None (*n* = 8)	At least one** (***n* = 15)	None (*n* = 18)
*Age group*				
18–34 years	1 (4.0)	0 (0)	1 (6.7)	0 (0)
35–49 years	4 (16.0)	1 (12.5)	4 (26.7)	1 (5.6)
50–64 years	11 (44.0)	2 (25.0)	4 (26.7)	9 (50.0)
≥ 65 years	9 (36.0)	6 (62.5)	6 (40.0)	8 (44.4)
*Highest educational level*				
Secondary modern school (Hauptschule)	7 (28.0)	1 (12.5)	5 (33.3)	3 (16.7)
Intermediate school/O-levels (Realschule)	8 (32.0)	2 (25.0)	4 (26.7)	6 (33.3)
A levels/university entrance qualification (Fachhochschulreife/Abitur)	9 (36.0)	5 (62.5)	6 (40.0)	8 (44.4)
*PHQ-2 summary score ≥ 3*	18 (72.0)	6 (75.0)	11 (73.3)	13 (72.2)
*GAD-2 summary score ≥ 3*	22 (88.0)	7 (87.5)	14 (93.3)	15 (83.3)
*Having close persons with an advanced or metastatic cancer in the past*	19 (76.0)	3 (37.5)	12 (80.0)	10 (55.6)
*Having obtained information regarding advanced or metastatic cancers*	14 (56.0)	3 (37.5)	9 (60.0)	8 (44.4)
*Patient with CCI ≥ 3 points*	4 (16.0)	2 (25.0)	3 (20.0)	3 (16.7)
*Patient’s menopausal status*				
Premenopausal	8 (32.0)	0 (0)	4 (26.7)	6 (33.3)
Perimenopausal	0 (0)	2 (25.0)	0 (0)	0 (0)
Postmenopausal	16 (64.0)	6 (75.0)	10 (66.7)	12 (66.7)
Male patient	1 (4.0)	0 (0)	1 (6.7)	0 (0)
*Patient with HR + tumour*	20 (80.0)	6 (75.0)	12 (80.0)	14 (77.8)
*Patient with HER2- tumour*	22 (88.0)	5 (62.5)	15 (100)	12 (66.7)
*Time since first diagnosis of metastasis in the patient ≥ 1 year*	11 (44.0)	3 (37.5)	7 (46.7)	7 (38.9)
*Type of mBC therapy of the patient*^$1^				
Chemotherapy	10 (40.0)	8 (100)	8 (53.3)	10 (55.6)
Endocrine therapy	10 (80.0)	4 (50.0)	12 (80.0)	12 (66.7)
Targeted therapy	7 (28.0)	3 (27.5)	4 (26.7)	6 (33.3)
Osteoprotective therapy	14 (56.0)	3 (37.5)	7 (46.7)	10 (55.6)
*Patient with severe depressive symptoms*	8 (32.0)	3 (37.5)	4 (26.7)	7 (38.9)
*Patient with severe anxiety symptoms*	5 (20.0)	1 (12.5)	4 (26.7)	2 (11.1)
*Patient with at least one unmet need*	16 (64.0)	3 (37.5)	9 (60.0)	10 (55.6)
*Patient with at least one information need*	13 (52.0)	2 (25.0)	9 (60.0)	6 (33.3)

### Assessment of helpfulness of a potential course or app for relatives

Regarding the helpfulness of certain contents of a potential course/app, a huge variation in the responses was observed (Table 5). Exercises for personal coping with the new situation and relaxation exercises were considered to be less to moderately helpful. However, most relatives would consider information on socio-legal issues very helpful.

**Table 5:** Assessment of helpfulness of a potential course or app for relatives (*n* = 33)

Information source	1 = not helpful at all	2	3	4	5	6 = very helpful
*Exercises for personal coping with the new situation*	10 (30.3)	1 (3.0)	6 (18.2)	6 (18.2)	4 (12.1)	10 (30.3)
*Relaxation exercises*	6 (18.2)	4 (12.1)	8 (24.2)	5 (15.2)	2 (6.1)	6 (18.2)
*Information on socio-legal issues*	1 (3.0)	4 (12.1)	5 (15.2)	7 (21.2)	13 (39.4)	1 (3.0)
*(missing n* = *1)*						

## Discussion

The aim of the present study was to investigate information and support needs in a German cohort of relatives of mBC patients. We found that three-quarters of the relatives had at least one unmet need, mostly observed in the categories “medical information about the relative’s illness” and “contact person who coordinates the treatment/aftercare”. Furthermore, almost half of the relatives had at least one information need, mostly observed in the categories “the likely course of the disease in the relative” and “how to deal with the treatment side effects”. These figures are comparable to those reported by relatives of patient receiving palliative care, such as metastatic cancers, in studies including patients with different types of cancer [11, 12]. In their systematic review, Lambert et al. (2012) also identified the unmet needs domains “information about the illness and treatment”, “coordination and continuity of care”, “assistance with patient daily needs”, and “access to health services”, among others [11]. Each of the domains was endorsed by up to a third of relatives. Comparably, Schmid-Büchi et al. (2008 & 2011) reported that more than half of the relatives of patients undergoing BC treatment needed information about the patients’ treatment [19, 20]. However, Lambert et al. (2012) acknowledged that the quantification and comparability of unmet needs is challenging, due to the wide variety of unmet needs measures and definitions that were employed [11].

Concerning potential influencing factors, we found that relatives in older age groups, with higher educational levels, were less likely to report unmet needs. Lambert et al. (2012) found diverging results regarding age and educational levels [11]. In only four of 15 articles, older age and higher educational levels were associated with fewer unmet needs. Other factors associated with higher unmet needs included being female, not being the spouse of the patient, or having lower social support. However, we did not include these factors in our study.

In our study, relatives who had close persons with an advanced or metastatic cancer in the past, who obtained information regarding advanced or metastatic cancers, if the patients had HER2- tumours, and if the patients had at least one unmet or information need, were more likely to report unmet or information needs compared to their respective counterparts. The procurement of information pertaining to advanced or metastatic cancers may give rise to further inquiries, particularly with regard to potential treatments and aftercare. A similar phenomenon may occur in the case of relatives of patients who have unmet information needs. The reasons why patients with HER2- tumours often have unmet support or information needs, despite having a notably improved prognosis compared to patients with HER2 + tumours, remain unclear [21]. Patients’ QoL was not found to be associated with their relatives’ unmet or information needs. As reported by Hodgkinson et al. (2007), discrepancies between patients’ and their partners’ needs can occur [22].

The large proportion of relatives with unmet information needs raises further questions, given that relatives often accompany BC patients to appointments and usually receive the same information. The desire for additional information, or the perception of not having received all the available information, may be a coping mechanism in view of their burdensome situation. Indeed, information-seeking as a coping strategy has been observed in patients with various chronic diseases and their relatives [23–25]. Targeting these coping strategies of relatives of mBC patients in dedicated support programmes may be important to improve their QoL.

A further aim of the study was to assess the QoL of the relatives and to analyse the relation to their information and support needs. In 72.7% and 88.0% of the relatives in our cohort, there was evidence for severe depressive or anxiety symptoms, respectively. For comparison, among male participants of the Mental Health Surveillance Study conducted by the Robert Koch Institute (representative of the general male population in Germany), only 19% and 11% reported moderate to severe depressive and anxiety symptoms, respectively [16, 17]. However, we did not find an association between unmet needs and depressive or anxiety symptoms. A lower prevalence of anxiety and depression was found in studies including relatives of patients with newly diagnosed BC or BC survivors [19, 22]. Due to the severity of the disease and the predicted life expectancy, relatives of patients with mBC may be more affected by those symptoms. Other studies showed contrasting results. Agustina et al. (2023) found that early palliative care was associated with reduced psychological distress and burden among caregivers or partners [26]. However, Wagner et al. (2006) reported that disease stage was not associated with QoL in partners [27]. Several studies identified a high prevalence of distress in relatives of patients with cancer and found it to be associated with their unmet needs [11, 19, 22]. Yet we did not include questionnaires on distress in our study.

It was acknowledged that there remains a paucity of knowledge surrounding relatives’ needs and the manner in which women’s and partners’ health and emotional distress may influence the interaction between patients and their partners [20]. The paucity of longitudinal studies is particularly salient, given the dearth of knowledge about how unmet needs change over time [11]. In the present study, mBC patients’ relatives stated that they would acknowledge a potential app or course providing information on socio-legal issues. Furthermore, healthcare professionals may be pivotal in facilitating communication between patients and their relatives, playing a crucial role in encouraging patients to involve their family members in the disease and treatment process. Informal caregivers, such as relatives of patients with mBC, often lack desired training, have limited resources, and high additional demands [7]. Dedicated support programmes, targeting different aspects of psychosocial interventions for relatives of patients with breast cancer, should be developed in cancer clinics [7, 19, 28]. These programmes could include communication-based interventions, coping skills training interventions, multicomponent interventions, and stress reduction interventions [28, 29]. Specifically, a systematic review found that interventions focusing on problem-solving and communication skills yielded the largest effects on QoL of cancer patient caregivers [29]. However, it should be noted that most caregivers in this review were female. Hence, these interventions may not be equally effective for relatives of mBC patients, who are predominantly male.

Our study has several strengths and limitations. To the best of our knowledge, this was the first study evaluating unmet support and information needs in a German cohort of relatives of mBC patients. As previously described, our mBC cohort is comparable to other (m)BC patients in terms of expression of HR and HER2 status, and metastatic sites [18, 30–34]. Furthermore, in accordance with the findings of other studies on the needs of cancer patients’ relatives, all relatives were either spouses/partners, sons/daughters, brothers/sisters, sons-in-laws/daughters-in-laws, or parents [11]. However, our study cohort was younger compared to most mBC patients in the literature [35, 36]. This may indicate that the patients in our cohort may be characterised by more aggressive tumours [37]. Last, a major limitation results from the small sample size, limiting the generalisability of the results for relatives of mBC patients. Furthermore, the possibility of attrition bias cannot be discounted, as more engaged patients with more engaged relatives may have participated in the study.

## Conclusion

Relatives of mBC patients frequently reported unmet and information needs, as well as anxiety and depressive symptoms. Dedicated support programmes for relatives of patients’ mBC should be developed, targeting their unmet and information needs regarding mBC, treatments, side effects, prognosis, and aftercare. Furthermore, physicians should be aware of actively involving mBC patients’ relatives in the disease and treatment process.

## Data Availability

Data are available on request to the authors.

## Abbreviations

BC: Breast cancer
mBC: Metastatic breast cancer
CCI: Charlson comorbidity index
eCRF: Electronic case report form
GAD-2: Generalized Anxiety Disorder 2
HER2: Human epidermal growth factor receptor 2
HR: Hormonal receptor
PHQ-4: Patient Health Questionnaire 4
QoL: Quality of life

## References

[1] Cardoso F, Harbeck N, Fallowfield L, Kyriakides S, Senkus E (2012) Locally recurrent or metastatic breast cancer: ESMO clinical practice guidelines for diagnosis, treatment and follow-up. Ann Oncol 23(Suppl 7):vii11-19 22997442 10.1093/annonc/mds232

[2] Hattori M, Iwata H (2018) Advances in treatment and care in metastatic breast cancer (MBC): are there MBC patients who are curable? Chin Clin Oncol 7(3):23 29860850 10.21037/cco.2018.05.01

[3] Leitlinienprogramm Onkologie (Deutsche Krebsgesellschaft, Deutsche Krebshilfe, AWMF): S3-Leitlinie Früherkennung, Diagnose, Therapie und Nachsorge des Mammakarzinoms. In*.*, vol. Version 4.3; 2020.

[4] Pitceathly C, Maguire P, Haddad P, Fletcher I (2004) Prevalence of and markers for affective disorders among cancer patients’ caregivers. J Psychosoc Oncol 22(3):45–68

[5] Turner D, Adams E, Boulton M, Harrison S, Khan N, Rose P, Ward A, Watson EK (2013) Partners and close family members of long-term cancer survivors: health status, psychosocial well-being and unmet supportive care needs. Psychooncology 22(1):12–19 21905160 10.1002/pon.2050

[6] Northouse LL, Katapodi MC, Schafenacker AM, Weiss D (2012) The impact of caregiving on the psychological well-being of family caregivers and cancer patients. Semin Oncol Nurs 28(4):236–245 23107181 10.1016/j.soncn.2012.09.006

[7] van Ryn M, Sanders S, Kahn K, van Houtven C, Griffin JM, Martin M, Atienza AA, Phelan S, Finstad D, Rowland J (2011) Objective burden, resources, and other stressors among informal cancer caregivers: a hidden quality issue? Psychooncology 20(1):44–52 20201115 10.1002/pon.1703 PMC4479404

[8] Sharpe L, Butow P, Smith C, McConnell D, Clarke S (2005) The relationship between available support, unmet needs and caregiver burden in patients with advanced cancer and their carers. Psychooncology 14(2):102–114 15386783 10.1002/pon.825

[9] Weitzner MA, McMillan SC, Jacobsen PB (1999) Family caregiver quality of life: differences between curative and palliative cancer treatment settings. J Pain Symptom Manage 17(6):418–428 10388247 10.1016/s0885-3924(99)00014-7

[10] Sepúlveda C, Marlin A, Yoshida T, Ullrich A (2002) Palliative care: the World Health Organization’s global perspective. J Pain Symptom Manage 24(2):91–96 12231124 10.1016/s0885-3924(02)00440-2

[11] Lambert SD, Harrison JD, Smith E, Bonevski B, Carey M, Lawsin C, Paul C, Girgis A (2012) The unmet needs of partners and caregivers of adults diagnosed with cancer: a systematic review. BMJ Support Palliat Care 2(3):224–230 24654195 10.1136/bmjspcare-2012-000226

[12] Friðriksdóttir N, Saevarsdóttir T, Halfdánardóttir S, Jónsdóttir A, Magnúsdóttir H, Olafsdóttir KL, Guðmundsdóttir G, Gunnarsdóttir S (2011) Family members of cancer patients: needs, quality of life and symptoms of anxiety and depression. Acta Oncol 50(2):252–258 21231786 10.3109/0284186X.2010.529821

[13] BRE-BY-MED / digiOnko. https://www.med.uni-wuerzburg.de/en/epidemiologie/forschung/projekte/versorgungsforschung/bre-by-med-digionko/. Accessed 13 Apr 2026

[14] Löwe B, Wahl I, Rose M, Spitzer C, Glaesmer H, Wingenfeld K, Schneider A, Brähler E (2010) A 4-item measure of depression and anxiety: validation and standardization of the Patient Health Questionnaire-4 (PHQ-4) in the general population. J Affect Disord 122(1–2):86–95 19616305 10.1016/j.jad.2009.06.019

[15] Faller H, Koch U, Brähler E, Härter M, Keller M, Schulz H, Wegscheider K, Weis J, Boehncke A, Hund B et al (2016) Satisfaction with information and unmet information needs in men and women with cancer. J Cancer Surviv 10(1):62–70 25956402 10.1007/s11764-015-0451-1

[16] Junker S, Damerow S, Walther L, Mauz E (2023) Development of a prototype for high-frequency mental health surveillance in Germany: data infrastructure and statistical methods. Front Public Health 11:1208515 37521976 10.3389/fpubh.2023.1208515 PMC10375021

[17] Robert-Koch-Institut (2024) Mental Health Surveillance—Beobachtung der psychischen Gesundheit der erwachsenen Bevölkerung in Deutschland. https://public.data.rki.de/t/public/views/hf-MHS_Dashboard/Dashboard?%3Aembed=y&%3AisGuestRedirectFromVizportal=y. Accessed 13 Apr 2026

[18] Brandstetter LS, Grau A, Heuschmann PU, Müller-Reiter M, Salmen J, Störk S, Wöckel A, Reese J-P (2025) Medication patterns and potentially inappropriate medication in patients with metastatic breast cancer: results of the BRE-BY-MED study. BMC Cancer 25(1):125 39844089 10.1186/s12885-025-13548-8 PMC11756166

[19] Schmid-Büchi S, van den Borne B, Dassen T, Halfens RJ (2011) Factors associated with psychosocial needs of close relatives of women under treatment for breast cancer. J Clin Nurs 20(7–8):1115–1124 21118322 10.1111/j.1365-2702.2010.03376.x

[20] Schmid-Büchi S, Halfens RJ, Dassen T, van den Borne B (2008) A review of psychosocial needs of breast-cancer patients and their relatives. J Clin Nurs 17(21):2895–2909 19012759 10.1111/j.1365-2702.2008.02490.x

[21] Prognostic and predictive factors in early, non-metastatic breast cancer. Edited by Vora SR. In: UpToDate. https://www.uptodate.com/contents/prognostic-and-predictive-factors-in-early-non-metastatic-breast-cancer?topicRef=744&source=see_link#H682640025. Accessed 13 Apr 2026

[22] Hodgkinson K, Butow P, Hunt GE, Wyse R, Hobbs KM, Wain G (2007) Life after cancer: couples’ and partners’ psychological adjustment and supportive care needs. Support Care Cancer 15(4):405–415 17043776 10.1007/s00520-006-0148-0

[23] Lillekroken D, Bye A, Halvorsrud L, Lundeby T (2025) Living with brain metastasis - a qualitative study of patients’ and family members’ coping strategies. Int J Qual Stud Health Well-being 20(1):2555228 40890980 10.1080/17482631.2025.2555228 PMC12406310

[24] Mason NF, Francis DB, Pecchioni LL (2022) Health information seeking as a coping strategy to reduce Alzheimer’s caregivers’ stress. Health Commun 37(2):131–140 32969294 10.1080/10410236.2020.1824665

[25] Paterson C, Roberts C, Li J, Chapman M, Strickland K, Johnston N, Law E, Bacon R, Turner M, Mohanty I et al (2024) What are the experiences of supportive care in people affected by brain cancer and their informal caregivers: a qualitative systematic review. J Cancer Surviv 18(5):1608–1629 37256499 10.1007/s11764-023-01401-5 PMC10229398

[26] Agustina R, Ispriantari A, Konlan KD, Lin MF (2023) Impact of early palliative care on the quality of life in caregivers of cancer patients: a systematic review. Worldviews Evid Based Nurs 20(3):178–190 36637053 10.1111/wvn.12629

[27] Wagner CD, Bigatti SM, Storniolo AM (2006) Quality of life of husbands of women with breast cancer. Psychooncology 15(2):109–120 15852406 10.1002/pon.928

[28] Kedia SK, Collins A, Dillon PJ, Akkus C, Ward KD, Jackson BM (2020) Psychosocial interventions for informal caregivers of lung cancer patients: a systematic review. Psychooncology 29(2):251–262 31701588 10.1002/pon.5271

[29] Waldron EA, Janke EA, Bechtel CF, Ramirez M, Cohen A (2013) A systematic review of psychosocial interventions to improve cancer caregiver quality of life. Psychooncology 22(6):1200–1207 22729992 10.1002/pon.3118

[30] American Cancer Society. Hormone therapy for breast cancer. https://www.cancer.org/cancer/types/breast-cancer/treatment/hormone-therapy-for-breast-cancer.html. Accessed 13 Apr 2026

[31] Clinical features, diagnosis, and staging of newly diagnosed breast cancer. In: UpToDate. Burstein H, Vora SR (eds) Waltham, MA: UpToDate, 2023. https://www.uptodate.com/contents/clinical-features-diagnosis-and-staging-of-newly-diagnosed-breast-cancer. Accessed 13 Apr 2026

[32] Rakha EA, Pinder SE, Bartlett JM, Ibrahim M, Starczynski J, Carder PJ, Provenzano E, Hanby A, Hales S, Lee AH et al (2015) Updated UK recommendations for HER2 assessment in breast cancer. J Clin Pathol 68(2):93–99 25488926 10.1136/jclinpath-2014-202571 PMC4316916

[33] Kennecke H, Yerushalmi R, Woods R, Cheang MC, Voduc D, Speers CH, Nielsen TO, Gelmon K (2010) Metastatic behavior of breast cancer subtypes. J Clin Oncol 28(20):3271–3277 20498394 10.1200/JCO.2009.25.9820

[34] Berman AT, Thukral AD, Hwang W-T, Solin LJ, Vapiwala N (2013) Incidence and patterns of distant metastases for patients with early-stage breast cancer after breast conservation treatment. Clin Breast Cancer 13(2):88–94 23218473 10.1016/j.clbc.2012.11.001

[35] Zentrum für Krebsregisterdaten im Robert Koch-Institut. Krebs in Deutschland für 2019/2020. In*.*, vol. 14. Ausgabe. Berlin; 2023.

[36] National Cancer Institute. Cancer statistics: fact sheets, cancer of the breast incidence and moortality, SEER incidence. Surveillance Epidemiology and End Results. https://seer.cancer.gov/statfacts/html/breast.html#incidence-mortality. Accessed 13 Apr 2026

[37] Zhang W, Wu S, Liu J, Zhang X, Ma X, Yang C, Cao M, Zhang S, Liu Y (2022) Metastasis patterns and prognosis in young breast cancer patients: a SEER database analysis. Front Oncol 12:872862 36313697 10.3389/fonc.2022.872862 PMC9608743

[38] digiOnko. https://www.digionko-bayern.de/. Accessed 13 Apr 2026

